# Analysis of Lsm1p and Lsm8p domains in the cellular localization of Lsm complexes in budding yeast

**DOI:** 10.1111/j.1742-4658.2009.07080.x

**Published:** 2009-07

**Authors:** Martin A M Reijns, Tatsiana Auchynnikava, Jean D Beggs

**Affiliations:** Wellcome Trust Centre for Cell Biology, University of EdinburghUK

**Keywords:** Lsm1–7p, Lsm2–8p, nuclear localization, P-bodies, *Saccharomyces cerevisiae*

## Abstract

In eukaryotes, two heteroheptameric Sm-like (Lsm) complexes that differ by a single subunit localize to different cellular compartments and have distinct functions in RNA metabolism. The cytoplasmic Lsm1–7p complex promotes mRNA decapping and localizes to processing bodies, whereas the Lsm2–8p complex takes part in a variety of nuclear RNA processing events. The structural features that determine their different functions and localizations are not known. Here, we analyse a range of mutant and hybrid Lsm1 and Lsm8 proteins, shedding light on the relative importance of their various domains in determining their localization and ability to support growth. Although no single domain is either essential or sufficient for cellular localization, the Lsm1p N-terminus may act as part of a nuclear exclusion signal for Lsm1–7p, and the shorter Lsm8p N-terminus contributes to nuclear accumulation of Lsm2–8p. The C-terminal regions seem to play a secondary role in determining localization, with little or no contribution coming from the central Sm domains. The essential Lsm8 protein is remarkably resistant to mutation in terms of supporting viability, whereas Lsm1p appears more sensitive. These findings contribute to our understanding of how two very similar protein complexes can have different properties.

*Saccharomyces cerevisiae* has at least two different heteroheptameric Sm-like (Lsm) complexes. The exclusively nuclear Lsm2–8p complex consists of the Lsm2 to Lsm8 proteins and forms the core of the spliceosomal U6 small nuclear ribonucleoprotein particle (snRNP) [1,2]. It is required for the stability [1–4] and nuclear localization [5] of U6 snRNA, as well as for pre-mRNA turnover [6]. In addition, various nuclear Lsm proteins interact with and/or are required for the processing of stable RNAs [7–12]. A second complex is formed by the Lsm1 to Lsm7 proteins and localizes exclusively to the cytoplasm [13]. This Lsm1–7p complex promotes mRNA decapping by Dcp1p/Dcp2p and subsequent degradation by Xrn1p 5′- to 3′-exonuclease [14–17]. These and various other proteins involved in deadenylation, decapping and decay accumulate in cytoplasmic foci, termed processing bodies (P-bodies) [18,19]. Under conditions that warrant high levels of mRNA turnover such as osmotic shock or glucose starvation, P-bodies increase in number and size [20]. The exact function of the Lsm1–7p complex is still unknown, but it is thought to act as a chaperone, remodelling mRNPs at a step following deadenylation, thereby promoting decapping [16]. A recent report that Lsm1–7p has higher affinity for shortened poly(A) tails suggests that increased binding to partially deadenylated RNAs may initiate this process [21]. Lsm2–8p is similarly thought to act as a chaperone, promoting U4/U6 di-snRNP formation [3,22].

Not much is known about what makes these two closely related complexes localize to different subcellular sites. We previously showed that nuclear accumulation of Lsm2–8p depends on importin β/Kap95p [5] and Nup49p, and that nuclear exclusion of Lsm1–7p does not depend on Xpo1p [13], but existing information on localization determinants within these complexes is minimal. Complex formation itself seems to be essential for Lsm1p and Lsm8p to localize to P-bodies and nuclei, respectively, suggesting that sequences present in multiple subunits combine to act as localization signals. Human LSm4 was shown to lose its localization to P-bodies when mutations were introduced in residues that are predicted to be involved in complex formation [23], and in yeast, Lsm2p and Lsm7p fail to localize to P-bodies in cells deleted for *LSM1* [24]. In yeast, Lsm8p fails to accumulate in the nucleus when cells are depleted of Lsm2p or Lsm4p [13], and in mammalian cells, injected recombinant LSm8 localizes throughout the cell, whereas recombinant LSm2–8 accumulates in the nucleus [25]. Finally, it was recently shown that the C-terminal asparagine-rich domain of Lsm4p plays a role in Lsm1–7p P-body localization [26,27] and in P-body assembly [28], emphasizing the importance of residues outside Lsm1p and Lsm8p for the localization and function of these complexes.

In budding yeast, only one form of the homologous Sm complex exists; it forms the core of non-U6 spliceosomal snRNPs and accumulates in the nucleus. Like the Lsm complexes, the Sm complex consists of seven different subunits forming a donut shape [3,29]. The basic residues in the C-terminal protuberances of two of the yeast Sm complex subunits, SmB and SmD1 proteins, have been shown to form separate nuclear localization signals that are functionally redundant [30]. The human SmB, SmD1 and SmD3 proteins were shown to contain similar signals important for nuclear localization [31]. Yeast Lsm8p is most closely related to SmB, with its C-terminus also containing a high level of basic lysine residues. However, although deletion of most of the C-terminus abolishes nuclear accumulation of the N-terminally green fluorescent protein (GFP)-tagged mutant protein, simultaneous mutation of six of these residues to alanine does not significantly affect localization, nor does this domain suffice for nuclear accumulation when fused to GFP [13]. This suggests that the Sm and Lsm2–8p complexes may not share the same mechanism to effect their nuclear accumulation.

Tharun *et al.* [24] performed extensive mutational analysis of Lsm1p showing the importance of residues proposed to be involved in RNA binding and complex formation, and of the C-terminal region for the functional competence of the Lsm1–7p complex. Although complex formation was proposed to be essential, mutations in the putative RNA-binding residues did not significantly affect Lsm1–7p localization to P-bodies [24]. To investigate the requirement for different domains of the Lsm1 and Lsm8 proteins in their function and localization, we created a series of mutant and hybrid proteins. We deleted or exchanged their N- and/or C-terminal domains, exchanged the central Sm domains or, in the case of Lsm8p, made point mutations in putative RNA-binding residues.

We investigated the cellular localization of GFP-tagged versions of these proteins, as well as their ability to support growth. Besides clarifying the relative importance of different regions of the Lsm1 and -8 polypeptides for localization and viability, our study highlights the effect that epitope tagging can have on the functional competence of proteins, with some mutant proteins supporting viability when tagged on one end but not when tagged on the other. Most importantly, we show that, although none of the Lsm1p and Lsm8p domains is absolutely essential for P-body or nuclear localization, their contribution to proper localization varies. We find that the N-terminal domains have the biggest impact on localization, whereas the C-terminal domains seem to play a secondary role, with apparently no or little contribution of the central Sm domain beyond its role in complex formation. Because it is known that complex formation is essential for correct localization [13,24], it is likely that residues from the N- and/or C-terminal domains form a nuclear exclusion or localization signal in combination with parts of other Lsm proteins.

## Results

### Production of Lsm1p and Lsm8p hybrids and mutants

**Fig. 1:**
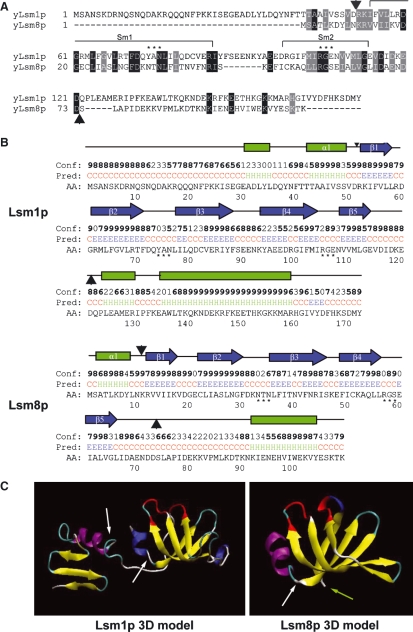
Structural features of Lsm1 and Lsm8 polypeptides. (A) Alignment of Lsm1p and Lsm8p using clustal w [48] (B) 2D structure predictions for Lsm1p and Lsm8p using 3D-PSSM [32]. Arrowheads indicate sites of N- and C-terminal deletions and fusions. * indicates residues forming putative RNA-binding Knuckle motifs. Green boxes indicate regions that are predicted to form α helices (H), blue arrows indicate regions that are predicted to form β strands (E) and lines indicate regions that are predicted to form random coil (C). Numbers indicate the confidence scores of these predictions for each residue, with 5–9 (in bold) indicating high confidence. (C) 3D structural prediction for Lsm1p and Lsm8p, made using default settings of swiss-model [49]. The model shown for Lsm1p covers residues 44–155 and is based on homology to a Sm-like archaeal protein from *Pyrobaculum aerophilum* (1m5q) [40]. The model shown for Lsm8p covers residues 1–67 and is based on homology to a heptameric Sm protein from *P. aerophilum* (1i8f) [50]. Arrows indicate break-points for our hybrids; the green arrow for Lsm8p indicates residue 67, whereas the break-point for our hybrids is residue 73; putative RNA-binding residues are shown in red.

In order to determine which regions of Lsm1p and Lsm8p should be tested by deletion or fusion in hybrid polypeptides, their amino acid (aa) sequences were aligned (Fig. 1A), and the 2D structural features analysed using the online 3D-PSSM server (Fig. 1B) [32]. The Lsm1 and Lsm8 polypeptides are most similar in the regions of the Sm1 and Sm2 motifs. These motifs form the Sm-fold, the hallmark of the Sm-like proteins, consisting of a five-stranded anti-parallel β sheet which is involved in intersubunit and protein–RNA contacts [29,33,34]. Crystal structures and cross-linking data have shown that RNA-binding residues in Sm(-like) proteins are located in loop 3 (between β2 and β3, i.e. the Sm1 motif) and loop 5 (between β4 and β5, i.e. the Sm2 motif) [35–38]. The consensus sequences for these so-called Knuckle motifs in eukaryotic Sm and archaeal Sm-like proteins are [His/Tyr]–Met–Asn for Knuckle I and Arg–Gly–Asp for Knuckle II [39]. It is not known how Lsm proteins bind RNA, but it is presumed to occur in a similar fashion. Putative RNA-binding residues for budding yeast Lsm1p and Lsm8p are indicated by asterisks in Fig. 1A,B, and in red in Fig. 1C.

**Fig. 2:**
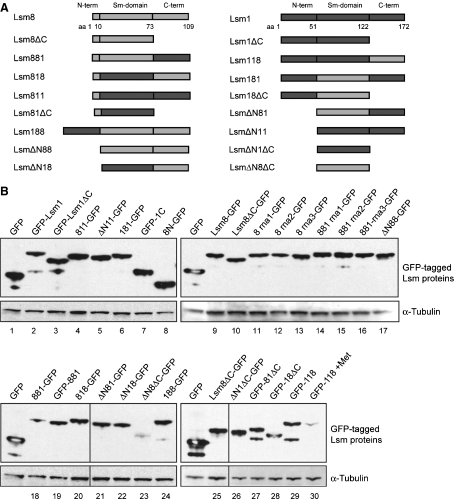
Lsm1p and Lsm8p mutant and hybrid proteins are stably produced. (A) Schematic overview of hybrids and deletion mutants of Lsm1p and Lsm8p. (B) MPS26 cells with plasmids expressing GFP-tagged hybrid and mutant proteins (Table S1) were grown in SD–Ura–Met (or SD–Ura+Met; lane 31) and aliquots of total protein from equal *D*_600_ units of cells were separated by SDS/PAGE and western blotted, probing with anti-GFP IgG2a. Hybridization with anti-(α-tubulin) IgG1 assesses equivalence of loading. Lsm8 *rna* mutants carry point mutations in putative RNA-binding residues (for details of all mutants and hybrids see Table S1). Additional bands in lanes 27 and 29 likely represent cleaved off GFP.

Prediction of secondary structures outside the Sm motifs reveals an α-helical region directly upstream of β1, which is another common feature of the Sm-fold (Fig. 1B). In addition, both proteins show potential α-helical structures in their C-terminal extensions, although a different 3D prediction for Lsm1p based on homology to an Sm-like archaeal protein from *Pyrobaculum aerophilum* (1m5q) [40] shows three anti-parallel β sheets in addition to a short α helix in the C-terminus of Lsm1p (Fig. 1C). Despite the differences between these models, both show a structured Lsm1p C-terminus, whereas most of the N-terminal extension of Lsm1p is predicted to be unstructured. Based on alignment and structure predictions, we define the N-terminal domain of Lsm1p as aa 1–51 and that of Lsm8p as aa 1–10 for the purpose of this study. The C-terminal domain of Lsm1p is defined as aa 122–172 and that of Lsm8p is aa 74–109, with the remaining residues representing the central Sm domains (Figs 1 and 2A). Fusions and deletions of the N- and C-terminal domains were thus designed to avoid disruption of the highly conserved Sm domain and other structured regions. All constructs used in this study are described in Table S1, and many are represented schematically in Fig. 2A.

Western analysis on total protein from cells expressing GFP-tagged versions of these hybrid and mutant polypeptides expressed from the *MET25* promoter shows that all except LsmΔN8ΔC–GFP (Fig. 2B, lane 23) were present at similar levels, indicating that they are stably expressed. In contrast to LsmΔN8ΔC–GFP, the central domain of Lsm1p, LsmΔN1ΔC–GFP (lane 26), is stably expressed. Lsm1p has a seven amino acid linker between the Sm1 and Sm2 motifs, which Lsm8p lacks. This may help it to form a more stable fold and/or may make it interact more strongly with its neighbours.

### N- and C-terminal domains do not suffice as localization signals

**Fig. 3:**
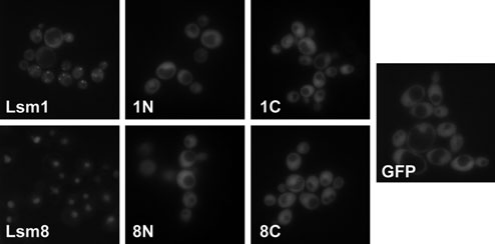
Lsm1p and Lsm8p N- and C-terminal extensions do not suffice for localization of GFP to P-bodies or nuclei. Strain MPS26 was transformed with pGFP–N-LSM1 (Lsm1), pMR144 (1N), pMR133 (1C), pGFP–N-LSM8 (Lsm8), pMR132 (8N), pMR156 (8C) or pGFP–N-FUS (GFP; Table S1). Cells were grown in SD–Ura–Met and localization was examined during log phase growth or 10 min after hypo-osmotic shock (for Lsm1p only). The images shown in this and all other figures are representative of the majority of cells in each given experiment.

The N- and C-terminal extensions of Lsm1p and Lsm8p were fused to the N- or C-terminus of GFP, respectively, in order to test whether they contain localization signals. Localization of each GFP-fusion was examined in live cells during log phase growth and after hypo-osmotic shock, and all were identical to that of GFP alone, i.e. throughout the cell, excluding vacuoles (Fig. 3 and data not shown). This indicates that the terminal extensions of Lsm1p and Lsm8p by themselves do not suffice as localization signals. This does not rule out that they may play a role in localization, possibly as part of a signal sequence together with contributions from other Lsm proteins.

### No single domain of Lsm8p is required absolutely for nuclear accumulation, although the N- and C-termini do contribute

To test whether the N- or C-terminal domain is essential for nuclear accumulation of Lsm8p, they were deleted or replaced with those of Lsm1p, creating Lsm8ΔCp, Lsm881p, LsmΔN88p and Lsm188p. Deletion of the central Sm domain was previously shown to abolish nuclear accumulation of Lsm8p, but this is most likely because of a loss of complex formation [13]. Therefore, to test whether this domain is essential for nuclear localization it was replaced with that of Lsm1p in Lsm818p, and the Sm domain of Lsm1p was replaced with that of Lsm8p in Lsm181p. Localization of these mutant proteins GFP-tagged at the N- or C-terminus was examined in live cells.

**Fig. 4:**
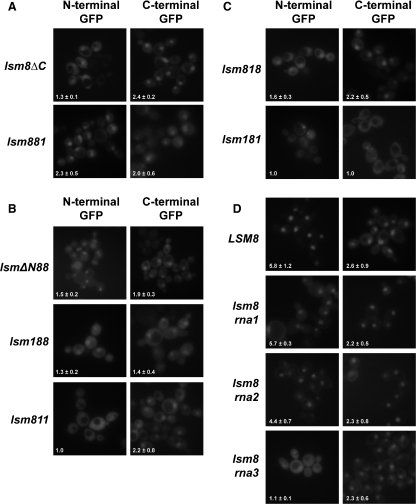
Effects of mutations in Lsm8p on its nuclear localization. (A) Lsm8p C-terminal domain mutations. (B) Lsm8p N-terminal domain mutations and recombinant Lsm1p containing the Lsm8p N-terminal 10 amino acids. (C) Sm domain replacements; see Fig 2A for an explanation of the constructs. (D) Mutations in or near the putative RNA-binding residues of Knuckle I and II. MPS26 was transformed with plasmids (A) pMR70, pMR80, pMR84 and pMR104; (B) pMR117, pMR126, pMR140, pMR141, pMR115 and pMR124; (C) pMR114, pMR116, pMR123 and pMR125; and (D) pMPS8, pMR76, pMR77, pMR78, pMR83, pMR92, pMR93 and pMR94 (see Table S1 for plasmid descriptions). Cells were grown in SD–Ura(–Met) and localization was examined in live cells during log-phase growth. We show the results for live cells only because we found that nuclear localization of our GFP-tagged proteins, including that of GFP–Lsm8, was significantly reduced after fixing (either using 4% formaldehyde or methanol). Intensities of nuclear and cytoplasmic signals were measured using imagej 1.38w and the average ratios of nuclear/cytoplasmic signals are indicated within each image. Where no nuclear accumulation was detected, a ratio of 1.0 is given.

The C-terminal domain of Lsm8p is not essential for nuclear accumulation because both Lsm8ΔCp and Lsm881p accumulate in the nucleus (Fig. 4A). However, compared with GFP–Lsm8 (Fig. 4D), both show increased cytoplasmic staining (the extent of which depends strongly on the placement of the tag), suggesting that the Lsm8p C-terminal domain does contribute to efficient nuclear localization. The N-terminal domain of Lsm8p is not required absolutely for nuclear accumulation, because both LsmΔN88p and Lsm188p accumulate in the nucleus (Fig. 4B). However, reduced nuclear and increased cytoplasmic localization, particularly for Lsm188p, suggests that the Lsm8p N-terminal domain contributes to nuclear accumulation and that the Lsm1p N-terminal domain likely favours cytoplasmic localization. This is confirmed with Lsm811p, which has only the N-terminal 10 amino acids and no other part of Lsm8p, and shows nuclear accumulation, at least when tagged at the C-terminus (Fig. 4B). Finally, nuclear localization of Lsm818–GFP and failure of Lsm181p to accumulate in the nucleus suggests that the Sm domain of Lsm8p is neither essential nor sufficient for nuclear accumulation (Fig. 4C).

We cannot rule out that some of our observations are caused by effects on complex stability. For example, loss of nuclear accumulation of N-terminally tagged mutant Lsm8 proteins may either be caused by masking of (part of) a localization signal, or by reduced complex formation because of steric hindrance by the N-terminal GFP tag. However, the first 20 amino acids of Lsm8p allow for increased nuclear localization when replacing the N-terminus of Lsm1p, suggestive of a more direct role for these residues in localization.

### Effect of RNA-binding mutations on Lsm8p nuclear localization

Three different mutations were created in putative RNA-binding residues in Lsm8p: *lsm8 rna1* (N28A, D31A) and *lsm8 rna2* (T34A, N35A) in or near the Knuckle I motif, and *lsm8 rna3* (R57A, G58W, S59A) in the Knuckle II motif. Based on analogous residues in Lsm1p (Fig. 1) [24] these would be expected to form the RNA-binding pocket (T34, N35, R57, S59) or to be important for the positioning of these residues (D31, G58). Mutation of putative RNA-binding residues in Lsm1p affected both mRNA decay and mRNA 3′-end protection, but not localization to P-bodies [24]. The *rna1* and *rna2* mutations did not significantly affect nuclear accumulation of Lsm8p (Fig. 4D). By contrast, N-terminally tagged Lsm8p carrying the *rna3* mutation failed to accumulate in the nucleus. However, the same protein tagged on the C-terminus accumulated in the nucleus at levels comparable with wild-type GFP-tagged Lsm8p. When, in addition to the *rna* mutations, the C-terminal domain of Lsm8p was replaced with that of Lsm1p (variants of Lsm881p) all proteins failed to accumulate in the nucleus, irrespective of which side the GFP tag was on (Fig. S1). This contrasts with Lsm881p lacking *rna* mutations (Fig. 4A), and suggests that mutations in and around the Knuckle motifs have a weak effect on Lsm8p localization, which becomes more apparent when combined with other mutations. We note that the fluorescence was very weak for the Lsm881 proteins with *rna* mutations despite seemingly unaffected expression levels (Fig. 2, lanes 11–16). We cannot rule out that loss of nuclear accumulation is indirect, through reduced complex formation.

### No single domain of Lsm1p is required absolutely for P-body localization, although the N-terminus does contribute

**Fig. 5:**
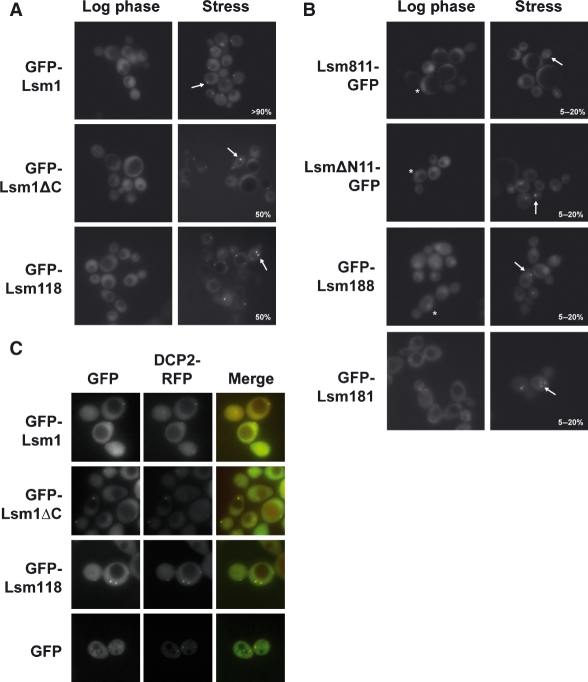
No single Lsm1p domain is essential for localization to P-bodies. (A) Lsm1p C-terminal domain mutations. (B) Lsm1p N-terminal domain mutations or central Sm domain replacement. See Fig 2A for an explanation of the constructs. Arrows indicate P-bodies; * indicate nuclear accumulation. (C) Lsm1p, Lsm1ΔCp and Lsm118p colocalize with Dcp2p in foci. AEMY25 (*lsm1Δ*) was transformed with plasmids: (A) pGFP–N-LSM1, pMR69 and pMR79; (B) pMR124, pMR126, pMR135 and pMR123; (C) pGFP–N-LSM1, pMR69, pMR79 or pGFP–N-FUS together with pRP1155 (DCP2–RFP; see Table S1 for plasmid descriptions). Cells were grown in SD–Ura(–Met) (A,B) or SD–Ura–Leu–Met (C) and localization was examined in live cells during log phase growth, after hypo-osmotic shock (stress; A,B) or after glucose starvation (C). Approximate percentages of cells showing focal accumulation of GFP signals after stress are given with each of the images in (A) and (B).

Because Lsm1p localizes exclusively to the cytoplasm [13] it seems likely that it has a nuclear exclusion signal that is formed either by its own residues or in combination with other Lsm1–7p subunits. GFP-tagged Lsm1p localizes throughout the cell, excluding vacuoles (Figs 3 and 5A), when expressed from the *MET25* promoter in our constructs, making it difficult to directly identify a nuclear exclusion signal. Because Lsm1–7p concentrates in P-bodies under stress conditions, we investigated whether any Lsm1p domain is required for localization to these foci. We tested deletion of the N- and/or C-terminal domains or replacement of the N-, C-terminal or Sm domains by those of Lsm8p in live *lsm1Δ* cells during log phase growth or under stress conditions.

The Lsm1p C-terminal domain is not absolutely required for P-body localization because Lsm1ΔCp and Lsm118p localize to P-bodies under stress conditions (Fig. 5A). The N-terminal domain is not essential either, nor is the central Sm domain, because Lsm811p, LsmΔN11p and Lsm181p localize to cytoplasmic foci under stress conditions (Fig. 5B). Localization of these hybrid proteins to P-bodies was reduced, however, because only 5–20% of cells expressing Lsm811p, LsmΔN11p or Lsm181p showed foci, compared with up to 50% of cells expressing Lsm1ΔCp or Lsm118p and > 90% of cells with GFP–Lsm1. Notably, Lsm188p accumulates in cytoplasmic foci in 5–20% of cells under stress conditions (Fig. 5B), suggesting that the N-terminal domain of Lsm1p is sufficient in combination with the Sm and C-terminal domains of Lsm8p (i.e. presumably in the context of an Lsm complex) to allow concentration in P-bodies, albeit with low efficiency. It is likely that reduced incorporation of some of these mutant proteins into the Lsm1–7p complex explains, at least in part, the reduced accumulation in cytoplasmic foci. Accumulation of these mutant proteins in foci under stress conditions suggests that these foci are P-bodies. This is confirmed by colocalization of GFP–Lsm1, GFP–Lsm1ΔC and GFP–Lsm118 with Dcp2–RFP after glucose starvation (Fig. 5C).

### Lsm1p and Lsm8p N-terminal domains support distinct cellular localizations

**Fig. 6:**
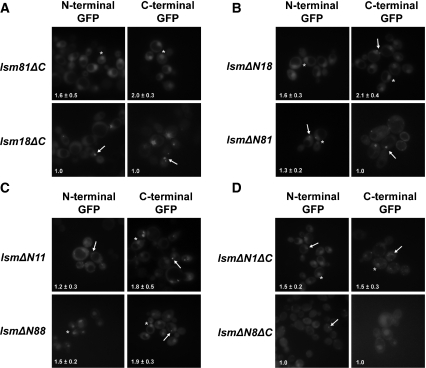
The Lsm1p and Lsm8p N-terminal domains are required for distinct localization. MPS26 was transformed with plasmids: (A) pMR129, pMR130, pMR137 and pMR138; (B) pMR143, pMR145, pMR147 and pMR148; (C) pMR134, pMR135, pMR140 and pMR141; (D) pMR150, pMR151, pMR153 and pMR154 (see Fig 2A for an explanation of the constructs and Table S1 for plasmid descriptions). Cells were grown in SD–Ura–Met to *D*_600_ = 1–2, and localization was examined in live cells. Nuclei are indicated by *, cytoplasmic foci are indicated by arrows. Intensities of nuclear and cytoplasmic signals were measured using imagej 1.38w and the average ratios of nuclear/cytoplasmic signals are indicated within each image. Where no nuclear accumulation was detected, a ratio of 1.0 is given.

Although both Lsm811p and Lsm188p localized to P-bodies in 5–20% of cells under stress conditions, in normal cells Lsm811p accumulated more in the nucleus and showed less cytoplasmic signal than did Lsm188p (Figs 4 and 5), suggesting that the N-terminal domains of Lsm1p and Lsm8p play a role in the localization to P-bodies and nuclei, respectively. A bigger change in the localization of mutant proteins with the N-terminal domain deleted compared with those with the C-terminal domain deleted is consistent with this (Figs 4 and 5). Hybrid proteins carrying the N-terminus of one protein and the Sm domain of the other localize according to the N-terminal contribution: Lsm81ΔCp shows nuclear accumulation and Lsm18ΔCp accumulates in cytoplasmic foci under stress conditions (Fig. 6A). Thus, in the absence of the C-terminal domain, the N-terminal domain, not the Sm domain, determines the subcellular localization. By contrast, LsmΔN18p and LsmΔN81p both show nuclear as well as focal accumulation (Fig. 6B), although the C-terminal contribution seems to determine the preferred site of localization: nuclear for LsmΔN18p and focal for LsmΔN81p, indicating that the C-terminal domain overrules any contribution of the Sm domain. Similarly, both LsmΔN11p and LsmΔN88p accumulate in the nucleus as well as in cytoplasmic foci (Fig. 6C), with more foci for the former and a higher level of nuclear accumulation for the latter, indicating that in the absence of an N-terminal domain distinct localization is lacking. Finally, the Lsm1p Sm domain by itself (LsmΔN1ΔCp) accumulates in both the nucleus and the cytoplasmic foci. The Lsm8p Sm domain shows extremely weak fluorescence, some of which localizes to vacuoles, no obvious nuclear accumulation and only very rare foci (Fig. 6D). Thus, in the absence of both N- and C-terminal extensions, the Sm domains of Lsm1p and Lsm8p do not have distinct subcellular localizations. The potential for P-body localization and nuclear accumulation of LsmΔN1ΔCp suggests incorporation into Lsm complexes, although this is likely to be reduced. Most N-terminal deletion mutants also showed some foci under normal growth conditions, whereas their number and intensity increased in the stationary phase or after hypo-osmotic stress (data not shown). This suggests that these mutant Lsm proteins lacking N-terminal domains may aggregate under normal growth conditions. It remains to be determined whether they aggregate as part of Lsm complexes or by themselves.

### Correlation between viability and correct localization

As a test of functional competence, at least in terms of essential processes, all mutant and hybrid proteins, either without a tag or GFP-tagged on the N- or C-terminus, were tested for their ability to support viability when produced under *P*_*MET25*_ control. The proteins were expressed in an *lsm1Δ* strain (AEMY25) or a strain with glucose-repressible *LSM8* (MPS11; *lsm8Δ* [*P*_*GAL*_*-HA-LSM8*]) and tested for growth at a range of temperatures by streaking on synthetic dropout medium (SD)–Ura (low level of expression) and SD–Ura–Met (high level of expression).

**Fig. 7:**
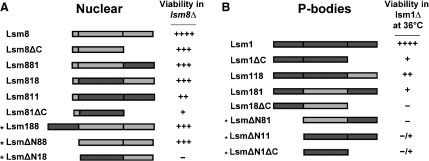
Correlation between viability and correct localization of Lsm1 and Lsm8 hybrid and mutant proteins. (A) Mutant proteins that accumulate in the nucleus. (B) Mutant proteins that accumulate in P-bodies. Viability was scored by comparison with the wild-type plasmid (++++) and the GFP only negative control (−). Proteins that accumulate both in nuclei and P-bodies are indicated by *. For a more detailed scoring of growth phenotypes for all different constructs see Tables S2 and S3.

We observed a positive correlation between viability in *lsm8Δ* and accumulation in the nucleus (Fig. 7A; Table S2). All mutant and hybrid proteins that showed nuclear accumulation supported viability, at least to some extent, whereas most of those that did not show nuclear accumulation did not support growth. Most mutants and hybrids supported growth better at lower (18 and 23 °C) than at higher (≥ 30 °C) temperatures, which suggests that Lsm2–8p complex stability may be reduced for many of them. In addition, most mutant and hybrid Lsm8 proteins with a GFP-tag on the Lsm8p N-terminus showed less growth than the same proteins with a C-terminal tag or with no tag, emphasizing the importance of a freely available Lsm8p N-terminus.

The stringency for growth at nonpermissive temperatures in the *lsm1Δ* background was higher, because few mutant and hybrid proteins supported growth at 36 or 37 °C (Fig. 7B and Table S3). Although not all mutant and hybrid proteins showing P-body accumulation supported growth at nonpermissive temperatures, all proteins that did support growth also accumulated in foci under stress conditions.

### Levels of mutant and hybrid proteins affect viability

We found that the levels of mutant and hybrid proteins had a significant effect on their ability to support growth. Whereas expression of wild-type Lsm1p and Lsm8p in the presence of 1mm methionine (i.e. the *MET25* promoter is repressed) allowed growth at all temperatures, most mutants and hybrids showed reduced viability. Northern analysis showed that in the presence and absence of 1 mm methionine the levels of *LSM8*–*GFP* mRNA expressed from *P*_*MET25*_ were, respectively, 3.5 and 15.5 times that of natively expressed *LSM8* mRNA (Fig. S2). The level of protein expression in the presence or absence of methionine showed a similar trend as is shown for GFP–Lsm118 in Fig. 2B (lanes 29 and 30). It is likely that many of the mutant and hybrid proteins would not support growth when expressed at normal levels, with higher protein levels driving complex formation and/or compensating for reduced protein stability.

### Lsm1p and Lsm8p localization determinants are poorly conserved

Amino acid sequences outside the Sm domains of Lsm1 and Lsm8 proteins are relatively poorly conserved from budding yeast to humans [3,24]. When the human homologues were expressed as GFP-fusion proteins in wild-type yeast cells, we observed considerable nuclear accumulation, but no significant focal accumulation after hypo-osmotic shock (Fig. S3 and data not shown). Expression of hLSm1 did not rescue temperature-sensitive growth of *lsm1Δ*, whereas hLSm8 allowed only minimal growth of *lsm8Δ* at 30 °C or below and only when expressed without a tag from the strong *ADH1* promoter. Thus, human LSm1 and LSm8 cannot efficiently substitute for the homologous yeast proteins. It is unclear what allows for their nuclear accumulation, but this suggests that they may incorporate into yeast Lsm complexes.

### Effects of mutant and hybrid proteins on Lsm complex formation and U6 snRNA association

**Fig. 8:**
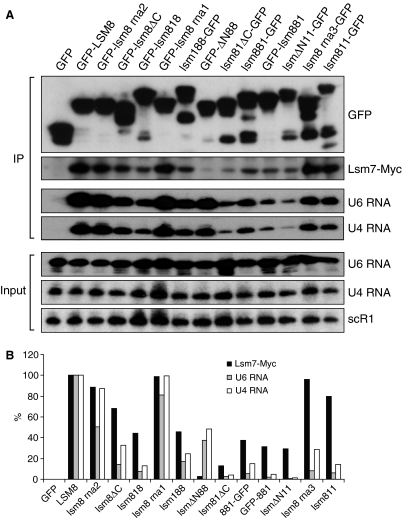
Analysis of complex formation and U6 snRNA binding of Lsm8 mutant and hybrid proteins. MPS26 cells carrying the appropriate plasmids were grown in SD–Ura–Leu–Met at 23 °C. Proteins were immunoprecipitated with affinity-purified rabbit anti-GFP. (A) Recombinant GFP-tagged protein and genomically encoded, co-precipitated Lsm7–Myc were visualized by western blotting; coprecipitated U4 and U6 snRNA, and total U6, U4 snRNA and scR1 present in the extracts were analysed by northern blotting. (B) Coprecipitated levels of Lsm7–Myc protein, U6 and U4 snRNA were quantified using imagequant software (Molecular Dynamics), normalized to GFP only background, and plotted as a percentage of wild-type. Immunoprecipitations were performed on two biological replicates, which showed similar results.

Reduced nuclear accumulation, as well as reduced viability, in strains expressing Lsm8 mutant and hybrid proteins may be caused indirectly by reduced Lsm complex formation. Reduced viability may also be caused by impaired U6 snRNA-binding ability of Lsm2–8p complex containing mutant or hybrid proteins. To investigate complex formation and U6 binding we performed immunoprecipitations using extracts from cells expressing GFP-tagged recombinant proteins that were able to support the growth of *lsm8Δ*. All recombinant proteins that were tested pull-down Lsm7p (Fig. 8), suggesting that all are able to incorporate into Lsm complexes, at least to some extent. Complex formation is not affected or only slightly reduced for the *rna* mutants, whereas Lsm8ΔCp and Lsm811p pull-down Lsm7p at > 70% of the wild-type level. Complex formation is reduced by > 50% for all other mutants, with LsmΔN88p most severely affected (3% of wild-type). U6 snRNA binding is reduced for all proteins tested, with binding least affected with the *rna1* mutant, whereas the *rna3* mutant shows severely reduced U6 binding despite almost normal complex formation. U6 snRNA binding is more strongly affected than complex formation for all mutant proteins with the exception of LsmΔN88p. This suggests that each of the Lsm8p domains contributes to proper U6 binding, either directly or indirectly, by affecting the RNA-binding ability of the resulting heteroheptameric Lsm complex (see Lsm8ΔC, Lsm818, Lsm881 and Lsm188). Minimal U6 binding by LsmΔN11p and Lsm811p, despite significant nuclear accumulation of these proteins, suggests that Lsm1–7p may have an intrinsically low affinity for U6 snRNA. LsmΔN88p binds U6 snRNA at almost 40% of wild-type levels despite strongly reduced complex formation. This means that either this protein can bind U6 without forming a complete heteroheptamer, or Lsm2–8p complexes are normally in excess over U6 snRNA. In the latter case, the Lsm2–ΔN88p complexes that do form may have normal affinity for U6 snRNA, but pull-down less because U6 is in excess over Lsm2–ΔN88p. U4 snRNA binding is less severely affected than U6 snRNA binding for all mutants, suggesting that a higher proportion of the mutant proteins are bound to the U4/U6 di-snRNP, than to the U6 snRNP, compared with wild-type.

### Effects of Lsm8 mutant and hybrid proteins on levels of U4 and U6 snRNAs

**Fig. 9:**
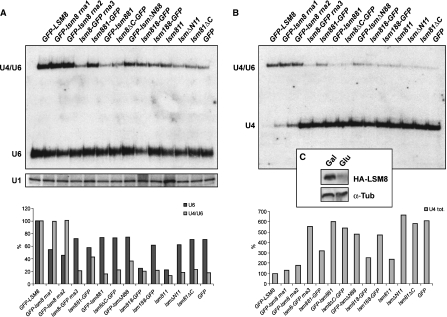
Levels of U4, U6 and U4/U6 snRNAs in *lsm8* mutant and hybrid strains. MPS11, which depends on a *CEN*–*HIS3* plasmid expressing HA–Lsm8p from the *GAL1-10* promoter, was transformed with plasmids expressing the mutant and hybrid proteins from the *MET25* promoter. Cells were grown in SDGal–Ura at 30 °C to mid-log phase before shifting them to SD–Ura–Met and growing for an additional 10 h at 30 °C. After 10 h equal numbers of cells were harvested for each of the mutants and total RNA was extracted under nondenaturing conditions. (A) Northern probed for U6 RNA after nondenaturing PAGE of total RNA. U1 snRNA was used as a loading control. (B) The same northern probed for U4 RNA. (C) Western blot on total protein probed with α-HA antibody confirms that cells are almost entirely depleted of HA–Lsm8p after 10 h of growth on glucose; similar levels of α-tubulin confirm equal loading. Quantifications on northern images were performed using imagequant; relative levels of U4, U6 and U4/U6 were corrected for U1 loading and expressed as a percentage of the level in the GFP–LSM8 positive control. Total U4 (U4 tot.) amounts, i.e. U4 present on its own as well as annealed to U6, were quantified in (B).

To investigate the extent to which these same Lsm8 mutant and hybrid proteins are able to stabilize U6 snRNA and promote formation of U4/U6 base-pairing, we analysed RNA extracted under nondenaturing conditions from *lsm8Δ* cells expressing these proteins. MPS11, which depends on a *CEN*–*HIS3* plasmid expressing HA–Lsm8p from the *GAL1-10* promoter, was transformed with plasmids expressing the mutant and hybrid proteins from the *MET25* promoter. Western analysis confirmed almost complete depletion of HA–Lsm8p after 10 h of growth on glucose (Fig. 9C). Northern analysis of U6 (Fig. 9A) and U4 snRNAs (Fig. 9B) after nondenaturing PAGE showed decreased levels of U4/U6 RNA for all the mutant and hybrid strains except *lsm8 rna1* and *lsm8 rna2*. In addition, levels of free U6 snRNA were decreased by ≈ 30–40% for all mutants and hybrids, including *lsm8 rna1*, *rna2* and the GFP only control. By contrast, levels of total U4 snRNA were significantly increased for all mutants and hybrids to levels two to six times that of wild-type. The increase was least for the *rna1* and *rna2* mutants, suggesting that the increase may be related to decreased levels of U4/U6 RNA. Thus, despite the ability of many of the mutant proteins to support growth, most do not protect U6 snRNA from degradation to the same extent as wild-type Lsm8p, nor do they allow for normal levels of U4/U6 di-snRNP formation (with the exception of *lsm8 rna1* and *rna2*). As shown in Fig. 8, this is the result of reduced complex formation and/or reduced U6 snRNA binding.

## Discussion

Here we show that the various domains of Lsm1p and Lsm8p contribute to different extents to their specific localization in the cell. The N-terminus, Sm domain and C-terminus of Lsm8p can be replaced with those of Lsm1p and still support viability, at least when moderately overexpressed from the *MET25* promoter. Although none of the Lsm8p domains is required absolutely in order for some level of nuclear accumulation to take place, each contributes to its exclusively nuclear localization. It seems that the N-terminal domain has the greatest effect on localization, the C-terminus plays a secondary role and the Sm domain probably only plays an indirect role by ensuring complex formation (e.g. Lsm818 accumulated in nuclei, whereas Lsm181 did not; Fig. 4C). Point mutations in putative RNA-binding residues within the Sm domain did not significantly affect nuclear localization.

Lsm1p seems to be more sensitive than Lsm8p to deletion or replacement of its domains, because few of the mutant and hybrid proteins supported growth at the nonpermissive temperature. Despite this, most still allowed for (some) accumulation in P-bodies under stress conditions, showing that none of the Lsm1p domains is required absolutely for this localization. However, a strong reduction in P-body localization of many of these mutant proteins shows that each of the domains does contribute to the accumulation in cytoplasmic foci. Similar to what we found for Lsm8p, there appears to be a hierarchy to the contribution of the Lsm1p domains to its localization. The N-terminus has the biggest effect on localization; the C-terminus plays a secondary role, determining the preferred localization in the absence of the N-terminus and the Sm domain may only contribute to localization through complex formation.

Mutant and hybrid Lsm1 and -8 proteins without the usual N-terminal domains showed some focal accumulation under normal growth conditions. Although at this point we have not formally ruled out the possibility that mutant proteins lacking the N-terminal domains are more prone to aggregation themselves, this raises the interesting possibility that the Lsm1p and Lsm8p N-termini prevent aggregation of the Lsm complexes, potentially by interacting with the prion-like C-terminal domain of Lsm4p. This asparagine-rich region of Lsm4p plays a role in Lsm1–7p accumulation in P-bodies [26,27], as well as in P-body assembly [28], and was recently shown to display many characteristics of a true prion protein [41]. It is plausible that the N-terminal domains of the neighbouring Lsm1 and -8 proteins could play a role in preventing aggregation of Lsm4p-containing complexes under normal growth conditions. Similarly, one could envisage a role for the Lsm1p N-terminus in the regulated accumulation of Lsm1–7p complexes in P-bodies under stress conditions. It may do so by effecting conformational change and/or post-translational modification of the Lsm4p C-terminus in response to stress.

Although apparently important for the specific subcellular localization of Lsm complexes, the N- or C-terminal domains of Lsm8p and Lsm1p are not by themselves sufficient for the nuclear localization of GFP or for its accumulation in P-bodies. This suggests a more complex localization signal that is likely to include sequences from other Lsm subunits, most likely the neighbouring Lsm2p and/or Lsm4p. Alternatively, Lsm1p and Lsm8p may affect the conformation of other subunits and/or of the entire complex, leading to nuclear accumulation or exclusion. Nuclear accumulation in budding yeast of GFP–hLSm1 and GFP–hLSm8, both of which lack a long N-terminal extension, and of hybrid and mutant proteins lacking the α1 helix suggests that Lsm complexes may localize to the nucleus by default. The longer budding yeast Lsm1p N-terminus is therefore likely to act as part of a nuclear exclusion signal. However, when we fused 36 or 49 residues of the yeast Lsm1p N-terminus to human LSm1 there was no significant decrease in its nuclear accumulation (data not shown).

Because stabilization of U6 snRNA was proposed to be the only essential function of the Lsm2 to Lsm8 proteins [42], the Lsm8p mutants and hybrids that support viability would be expected to bind and stabilize U6 snRNA. However, we found only a weak correlation between levels of U6 and U4/U6 RNA and cell viability, suggesting that additional functions of Lsm8p/Lsm2–8p may contribute to the growth phenotypes of the mutant strains. Interestingly, mutants that show reduced levels of U4/U6 also show increased levels of total U4 RNA. This was also observed for *lsm6Δ*, *lsm7Δ* and particularly *lsm5Δ* strains (our unpublished data), but only when analysing total cellular RNA levels, not when looking at RNA levels in splicing extracts [1,22]. It is unclear why defects in Lsm2–8p should lead to an increase in the total level of U4 snRNA, but, considering the importance of Lsm2–8p for recycling snRNPs [22], it may suggest higher stability of newly synthesized U4 snRNA compared with recycled U4 snRNA. Alternatively, Lsm2–8p may be more directly involved in processing and/or degradation of U4 snRNA.

Dissection of the Lsm1p and Lsm8p proteins has shown that their localization is not determined by any single feature, and has proved useful in determining the relative contributions of various domains for their localization. Further examination of the specific effects these mutants and hybrids may have on particular processes, for example, U4/U6 annealing, may further elucidate how the Lsm1–7p and Lsm2–8p complexes function.

## Materials and methods

### Yeast media, strains and plasmids

Yeast media and manipulations were as described previously [43]. To allow expression of wild-type, mutant and hybrid proteins from the *MET25* promoter of pGFP–N-FUS or pGFP–C-FUS plasmids [44], cultures were grown in SD lacking uracil and methionine. Wild-type *LSM1* and *LSM8* and deletion mutants were amplified by PCR and inserted into multiple cloning sites of pGFP–N-FUS or pGFP–C-FUS. Point mutations in *LSM8* were created using the Quikchange mutagenesis protocol (Stratagene, La Jolla, CA, USA). Hybrids were created by fusing the appropriate fragments using two-step PCR. All recombinants were checked by sequencing. Yeast strains used in this study are described in Table 1, and a list of plasmids is given in Table S1.

**Table 1:** Yeast strains used in this study.

Strain	Genotype	Reference
BMA38a	*MAT***a***ade2-1 his3Δ200 leu2-3,-112 trp1Δ1 ura3-1 can1-100*	B. Dujon, (Institut Pasteur, Paris, France)
AEMY25	*MAT***a***ade2-1 his3-11,-15 leu2-3,-112 trp1Δ1 ura3-1 lsm1Δ::TRP1*	[1]
MPS11	*MAT***a***ade2-1 his3-11,-15 leu2-3,-112 trp1*Δ*1 ura3-1 can1-100 lsm8Δ::TRP1 [pRS313, P*_*GAL*_*-HA-LSM8] LSM7:13myc-HphMX6*	[13]
MPS26	*MAT***a***ade2-1 his3-11,-15 leu2-3,-112 trp1*Δ*1 ura3-1 can1-100 lsm8Δ::TRP1 [pYX172] LSM7:13myc-HphMX6*	[13]

### Western blotting analysis of recombinant proteins

For crude protein extracts [45], three *D*_600_ units of yeast cells were lysed in 0.5 mL of 0.2 m NaOH on ice for 10 min, followed by trichloroacetic acid precipitation (final 5% w/v) for 10 min on ice. After centrifugation, the pellet was resuspended in 35 μL of dissociation buffer (0.1 m Tris/HCl pH 6.8, 4 mm EDTA, 4% SDS, 20% v/v glycerol, 2% v/v β-mercaptoethanol, 0.02% w/v bromophenol blue) and 15 μL of 1 m Tris base. Samples were heated at 95 °C for 10 min before separation by SDS/PAGE [14% gel; Acrylamide/Bis-Acrylamide (37.5 : 1 ratio) from Sigma]. Proteins were transferred to a poly(vinylidene difluoride) (PVDF) membrane and detected with mouse anti-GFP IgG2a (BD Bioscience, San Jose, CA, USA) or anti-(α-tubulin) IgG1 (Sigma, St Louis, MO, USA) and sheep-(anti-mouse IgG)–HRP (Amersham Biosciences, Piscataway, NJ, USA). To show depletion of HA–Lsm8p after 10 h growth in glucose (Fig. 8), total protein was similarly prepared from cells before and after 10 h growth in glucose, and the western blot was probed with HRP-conjugated mouse anti-HA IgG2a (Santa-Cruz Biotechnology, Santa Cruz, CA, USA).

### Fluorescence microscopy

Cells were grown at 30 °C in SD medium. To stress cells, cultures were centrifuged and cells were resuspended in water or medium lacking glucose. Live cells were examined by fluorescence microscopy using a Leica FW4000 fluorescence microscope. Images were captured using leica fw4000 software (Scanalytics, Fairfax, VA, USA) with a CH-250 16-bit, cooled CCD camera (Photometrics, Tucson, AZ, USA).

### RNA extraction and northern blotting

For analysis of levels of U4, U6 and U4/U6 RNA, total RNA was isolated under nondenaturing conditions [46]. Briefly, cells were grown to *D*_600_ = 0.5–1.0, ten *D*_600_ units were collected, washed with water and resuspended in 250 μL of RNA extraction buffer (100 mm LiCl, 1 mm EDTA, 100 mm Tris/Cl pH 7.5, 0.2% w/v SDS). Cells were broken in a cooled Thermomixer Comfort (Eppendorf, Cambridge, UK) by vigorous shaking for 15 min at 4 °C with 250 μL of phenol/chloroform (5 : 1, pH 4.7) and 100 μL of Zirconia beads (Ambion, Applied Biosystems, Warrington, UK). The aqueous phase was mixed with an equal volume of 2 × RNA loading buffer for separation by native PAGE (6%, 20 : 1, 0.5 × TBE). After northern blotting, the Hybond-N membrane (GE Healthcare, Chalfont St Giles, UK) was probed for U1, U4, U6 snRNA or scR1 RNA [27,47]. Northern blots were quantified using a STORM 860 phosphorimager and imagequant software (Molecular Dynamics, Sunnyvale, CA, USA). U1 was used as a loading control, with quantifications presented for U4, U6 and U4/U6 corrected for loading.

### Immunoprecipitations

Cells (MPS26 transformed with the appropriate plasmid; 500 mL) were grown at 23 °C to *D*_600_ = 0.6–0.7, centrifuged and snap frozen. To prepare extracts, cells were resuspended in 3 vol of lysis buffer (50 mm Hepes pH 7.5, 100 mm NaCl, 1 mm MgCl_2_, 0.3% Triton X-100, 1 mm dithiothreitol, Roche Complete protease inhibitor), and vortexed three times for 1 min with 200 μL of Zirconia beads. Extracts were clarified by centrifugation for 5 min at 1200 ***g*** and 10 min at 16 000 ***g***. Mouse anti-GFP IgG2a (Molecular Probes, Invitrogen, Carlsbad, CA, USA) was bound to Protein A dynabeads (Invitrogen). Beads were mixed with extracts and incubated for 1 h at 4 °C and washed with lysis buffer. For northern analysis, beads were phenol/chloroform extracted and resolved by denaturing PAGE (6%). For western analysis, beads were boiled in SDS loading buffer and separated by SDS/PAGE (4–12% gradient gel; Invitrogen), blotted and probed with HRP-conjugated anti-c-Myc IgG1 (Roche, Basel, Switzerland) or rabbit anti-GFP (N-terminal affinity purified; Sigma). Northern blotting, western blotting and quantifications were carried out as described above.

## Abbreviations

aa: amino acid(s)
GFP: green fluorescent protein
Lsm: Sm-like
P-bodies: processing bodies
SD: synthetic dropout medium
snRNP: small nuclear ribonucleoprotein particle

## Supporting information

The following supplementary material is available:

**Fig. S1.** Effects of mutations in putative RNA-binding residues of Lsm8p on nuclear localization of Lsm881p.

**Fig. S2.** Comparison of *LSM8-GFP* (expressed from *Pmet25*) and native *LSM8* transcript levels.

**Fig. S3.** Human LSm1 and LSm8 proteins accumulate in the nuclei of budding yeast cells.

**Table S1.** Plasmids used in this study.

**Table S2.** Viability of *lsm8Δ* expressing mutant and hybrid Lsm8 proteins.

**Table S3.** Viability at nonpermissive temperature of *lsm1Δ* expressing mutant Lsm1 proteins.

This supplementary material can be found in the online version of this article.
