# Supplementary material for: Analysis of Lsm1p and Lsm8p domains in the cellular localization of Lsm complexes in budding yeast
Source: FEBS J. 2009 Jul;276(13):3602–17. doi: 10.1111/j.1742-4658.2009.07080.x (PMC2776932; doi:10.1111/j.1742-4658.2009.07080.x)
Supplement: Supplementary file 1 [file ejb0276-3602-SD1.pdf]

SUPPLEMENTARY MATERIALS

Analysis of Lsm1p and Lsm8p domains in cellular localisation of Lsm complexes in budding yeast

Martin A. M. Reijns, **Tatsiana Auchynnikava** and Jean D. Beggs

Table S1. Plasmids used in this study

Plasmid	Description	Reference
pGFP-N-FUS	*CEN6*, *URA3* shuttle vector with MCS for N-terminal GFP tagging under control of *MET25* promoter	[1]
pGFP-C-FUS	*CEN6*, *URA3* shuttle vector with MCS for C-terminal GFP tagging under control of *MET25* promoter	[1]
p413-ADH1	*CEN6*, *HIS3* shuttle vector with MCS for expression from *ADH1* promoter, *CYC1* terminator	[2]
pYX172	2μ, *LEU2*, *SNR6*	[3]
pGFP-N-LSM1[a]	*LSM1* coding region in frame with N-terminal GFP tag	[4]
pMPS8	pGFP-N-LSM8 [a]	[5]
pMR69	pGFP-N-Lsm118 [a]	This work
pMR70	pGFP-N-Lsm881 [a]	This work
pMR73	pGFP-N-Lsm881 rna1 [a,d]: N28A and D31A mutations introduced into pMR70	This work
pMR74	pGFP-N-Lsm881 rna2 [a,d]: T34A and N35A mutations introduced into pMR70	This work
pMR75	pGFP-N-Lsm881 rna3 [a,d]: R57A, G58W and S59A mutations introduced into pMR70	This work
pMR76	pGFP-N-Lsm8 rna1 [a]: N28A and D31A mutations introduced into pGFP-N-LSM8	This work

Plasmid	Description	Reference
pMR77	pGFP-N-Lsm8 rna2 [a]: T34A and N35A mutations introduced into pGFP-N-LSM8	This work
pMR78	pGFP-N-Lsm8 rna3 [a]: R57A, G58W and S59A mutations introduced into pGFP-N-LSM8	This work
pMR79	pGFP-N-Lsm1ΔC [a,d]	This work
pMR80	pGFP-N-Lsm8ΔC [a,d]	This work
pMR83	pGFP-C-LSM8 [b]	[6]
pMR84	pGFP-C-Lsm881 [b,d]	This work
pMR86	pGFP-C-LSM1 [b,d]	This work
pMR87	pGFP-C-Lsm118 [b,d]	This work
pMR88	pMET25-LSM1 [c]	This work
pMR89	pMET25-Lsm118 [c,d]	This work
pMR90	pMET25-LSM8 [c,d]	This work
pMR91	pMET25-Lsm881 [c,d]	This work
pMR92	pGFP-C-Lsm8 rna1 [b] (N28A; D31A)	This work
pMR93	pGFP-C-Lsm8 rna2 [b] (T34A; N35A)	This work
pMR94	pGFP-C-Lsm8 rna3 [b] (R57A; G58W; S59A)	This work
pMR95	pGFP-C-Lsm881 rna1 [b] (N28A; D31A)	This work
pMR96	pGFP-C-Lsm881 rna2 [b] (T34A; N35A)	This work
pMR97	pGFP-C-Lsm881 rna3 [b] (R57A; G58W; S59A)	This work
pMR98	pMET25-Lsm8 rna1 [c] (N28A; D31A)	This work
pMR99	pMET25-Lsm8 rna2 [c] (T34A; N35A)	This work
pMR100	pMET25-Lsm8 rna3 [c] (R57A; G58W; S59A)	This work
pMR101	pMET25-Lsm881 rna1 [c,d] (N28A; D31A)	This work
pMR102	pMET25-Lsm881 rna2 [c,d] (T34A; N35A)	This work
pMR103	pMET25-Lsm881 rna3 [c,d] (R57A; G58W; S59A)	This work

Plasmid	Description	Reference
pMR104	pGFP-C-Lsm8ΔC [b,d]	This work
pMR105	pMET25-Lsm8ΔC [c,d]	This work
pMR114	pGFP-N-Lsm181 [a,d]	This work
pMR115	pGFP-N-Lsm811 [a,d]	This work
pMR116	pGFP-N-Lsm818 [a,d]	This work
pMR117	pGFP-C-Lsm188 [b,d]	This work
pMR119	pMET25-Lsm181 [c,d]	This work
pMR120	pMET25-Lsm811 [c,d]	This work
pMR121	pMET25-Lsm818 [c,d]	This work
pMR122	pMET25-Lsm188 [c,d]	This work
pMR123	pGFP-C-Lsm181 [b,d]	This work
pMR124	pGFP-C-Lsm811 [b,d]	This work
pMR125	pGFP-C-Lsm818 [b,d]	This work
pMR126	pGFP-N-Lsm188 [a,d]	This work
pMR129	pGFP-N-lsm81ΔC [a,d]	This work
pMR130	pGFP-C-lsm81ΔC [b,d]	This work
pMR131	pMET25-lsm81ΔC [c,d]	This work
pMR132	pGFP-C-lsm8N: *LSM8* N-terminus (aa1-10) cloned in frame with C-terminal GFP-tag in pGFP-C-FUS	This work
pMR133	pGFP-N-lsm1C: *LSM1* C-terminus (aa122-172) cloned in frame with N-terminal GFP-tag in pGFP-N-FUS	This work
pMR134	pGFP-N-lsmΔN11 [a,d]	This work
pMR135	pGFP-C-lsmΔN11 [b,d]	This work
pMR136	pMET25-lsmΔN11 [c,d]	This work
pMR137	pGFP-N-lsm18ΔC [a,d]	This work
pMR138	pGFP-C-lsm18ΔC [b,d]	This work

Plasmid	Description	Reference
pMR139	pMET25-lsm18ΔC [c,d]	This work
pMR140	pGFP-N-lsmΔN88 [a,d]	This work
pMR141	pGFP-C-lsmΔN88 [b,d]	This work
pMR142	pMET25-lsmΔN88 [c,d]	This work
pMR143	pGFP-N-lsmΔN18 [a,d]	This work
pMR144	pGFP-C-lsm1N: *LSM1* N-terminus (aa1-51) cloned in frame with C-terminal GFP-tag in pGFP-C-FUS	This work
pMR145	pGFP-C-lsmΔN18 [b,d]	This work
pMR146	pMET25-lsmΔN18 [c,d]	This work
pMR147	pGFP-N-lsmΔN81 [a,d]	This work
pMR148	pGFP-C-lsmΔN81 [b,d]	This work
pMR149	pMET25-lsmΔN81 [c,d]	This work
pMR150	pGFP-N-lsmΔN1ΔC [a,d]	This work
pMR151	pGFP-C-lsmΔN1ΔC [b,d]	This work
pMR152	pMET25-lsmΔN1ΔC [c,d]	This work
pMR153	pGFP-N-lsmΔN8ΔC [a,d]	This work
pMR154	pGFP-C-lsmΔN8ΔC [b,d]	This work
pMR155	pMET25-lsmΔN8ΔC [c,d]	This work
pMPS8C (pMR156)	pGFP-N-lsm8C: *LSM8* C-terminus (aa65-109) cloned in frame with N-terminal GFP-tag in pGFP-N-FUS	[6]
pMR184	p413-ADH1 with hLSm8 coding sequence	This work
pMR185	p413-ADH1 with hLSm1 coding sequence	This work
pMR186	pGFP-N-hLSm1 [a]	This work
pMR187	pGFP-N-hLSm8 [a]	This work
pMR188	pGFP-C-hLSm8 [b]	This work
pMR194	pGFP-N-yhLsm1.1 [a]: aa 1- 36 of yeast *LSM1* fused to full hLSm1 coding sequence	This work

Plasmid	Description	Reference
pMR195	pGFP-N-yhLsm1.2 [a]: aa 1- 49 of yeast *LSM1* fused to aa 14-133 of hLSm1	This work
pRP1155	*DCP2* with C-terminal RFP tag in *CEN*, *LEU* shuttle vector (gift from R. Parker)	[7]

[a)] pGFP-N-Lsm: Coding sequence of *LSM* wild-type gene, mutant or hybrid inserted in frame with N-terminal GFP-tag in pGFP-N-FUS

[b)] pGFP-C-Lsm: Coding sequence of *LSM* wild-type gene, mutant or hybrid inserted in frame with C-terminal GFP-tag in pGFP-C-FUS

[c)] pMET25-Lsm: Coding sequence of *LSM* wild-type gene, mutant or hybrid inserted in pGFP-C-FUS without C-terminal GFP-tag

[d)] LsmABC: A is N-terminus of Lsm1p (aa 1-51) or Lsm8p (aa 1-10); deletion of this region is indicated by ΔN; B is central region containing Sm domain of Lsm1p (aa 52-121) or Lsm8p (aa 11-73); C is C-terminus of Lsm1p (aa 122-172) or Lsm8p (aa 74-109); deletion of this region is indicated by ΔC

Table S2. Viability of *lsm8Δ* expressing mutant and hybrid Lsm8 proteins

Plasmids were transformed into MPS11 and tested for growth on SD-Ura and SD-Ura-Met at 18, 23, 30, 36 and 37˚C. Amount and rate of growth were compared to that for pGFP-N-LSM8 and pGFP-N-FUS and given a score: ++++ for growth like the positive control, – for background growth and +++, ++ or + for anything in between. n.t. = not tested

	Viability in MPS11 (*lsm8Δ*) background									
**Plasmid**	**18ºC**		**23ºC**		**30ºC**		**36ºC**		**37ºC**	
	**-U**	**-U-M**	**-U**	**-U-M**	**-U**	**-U-M**	**-U**	**-U-M**	**-U**	**-U-M**
**pGFP-N-LSM8**	++++	++++	++++	++++	++++	++++	++++	++++	++++	++++
**pGFP-C-LSM8**	++++	++++	++++	++++	++++	++++	++++	++++	++++	++++
**pMET25-LSM8**	++++	++++	++++	++++	++++	++++	++++	++++	++++	++++
**pGFP-N-Lsm881**	-	+	-	+	-	-	n.t.	n.t.	n.t.	n.t.
**pGFP-C-Lsm881**	+++	++++	++	+++	-	++++	-	-	-	-
**pMET25-Lsm881**	+++	++++	++	++++	-	++++	-	-	-	-
**pGFP-N-Lsm8 rna1**	++++	++++	++++	++++	++++	++++	-	+++	-	+++
**pGFP-C-Lsm8 rna1**	++++	++++	++++	++++	++++	++++	++++	++++	+	+++
**pMET25-Lsm8 rna1**	++++	++++	++++	++++	++++	++++	++++	++++	++++	++++
**pGFP-N-Lsm8 rna2**	++++	++++	++++	++++	++++	++++	-	+++	-	+++
**pGFP-C-Lsm8 rna2**	++++	++++	++++	++++	++++	++++	++++	++++	+	+++
**pMET25-Lsm8 rna2**	++++	++++	++++	++++	++++	++++	++++	++++	++++	++++
**pGFP-N-Lsm8 rna3**	-	+	-	+	-	-	n.t.	n.t.	n.t.	n.t.
**pGFP-C-Lsm8 rna3**	++	+++	++	+++	-	++	-	-	n.t.	n.t.
**pMET25-Lsm8 rna3**	++	+++	++	+++	-	++	-	-	n.t.	n.t.

	Viability in MPS11 (*lsm8Δ*) background									
**Plasmid**	**18ºC**		**23ºC**		**30ºC**		**36ºC**		**37ºC**	
	**-U**	**-U-M**	**-U**	**-U-M**	**-U**	**-U-M**	**-U**	**-U-M**	**-U**	**-U-M**
**pGFP-N-Lsm881 rna1**	n.t.	n.t.	n.t.	n.t.	-	-	n.t.	n.t.	n.t.	n.t.
**pGFP-C-Lsm881 rna1**	-	-	n.t.	-	-	-	-	-	n.t.	n.t.
**pMET25-Lsm881 rna1**	-	-	n.t.	-	-	-	-	-	n.t.	n.t.
**pGFP-N-Lsm881 rna2**	n.t.	n.t.	n.t.	n.t.	-	-	n.t.	n.t.	n.t.	n.t.
**pGFP-C-Lsm881 rna2**	-	-	n.t.	-	-	-	-	-	n.t.	n.t.
**pMET25-Lsm881 rna2**	-	-	n.t.	-	-	-	-	-	n.t.	n.t.
**pGFP-N-Lsm881 rna3**	n.t.	n.t.	n.t.	n.t.	-	-	n.t.	n.t.	n.t.	n.t.
**pGFP-C-Lsm881 rna3**	-	-	n.t.	-	-	-	-	-	n.t.	n.t.
**pMET25-Lsm881 rna3**	-	-	n.t.	-	-	-	-	-	n.t.	n.t.
**pGFP-N-Lsm8ΔC**	-	+	-	+	-	-	n.t.	n.t.	n.t.	n.t.
**pGFP-C-Lsm8ΔC**	+++	++++	++	++++	-	+++	-	-	n.t.	n.t.
**pMET25-Lsm8ΔC**	+++	++++	++	++++	++	++++	-	-	n.t.	n.t.
**pGFP-N-Lsm118**	n.t.	-	n.t.	-	n.t.	-	n.t.	n.t.	n.t.	n.t.
**pGFP-C-Lsm118**	n.t.	-	n.t.	-	n.t.	-	n.t.	n.t.	n.t.	n.t.
**pMET25-Lsm118**	n.t.	-	n.t.	-	n.t.	-	n.t.	n.t.	n.t.	n.t.
**pGFP-N-Lsm181**	n.t.	-	n.t.	-	n.t.	-	n.t.	n.t.	n.t.	n.t.
**pGFP-C-Lsm181**	n.t.	-	n.t.	-	n.t.	-	n.t.	n.t.	n.t.	n.t.
**pMET25-Lsm181**	n.t.	-	n.t.	-	n.t.	-	n.t.	n.t.	n.t.	n.t.
**pGFP-N-Lsm811**	-	++	-	++	-	-	n.t.	n.t.	n.t.	n.t.
**pGFP-C-Lsm811**	-	++	-	-	-	-	n.t.	n.t.	n.t.	n.t.

Viability in MPS11 (*lsm8Δ*) background										
**Plasmid**	**18ºC**		**23ºC**		**30ºC**		**36ºC**		**37ºC**	
	**-U**	**-U-M**	**-U**	**-U-M**	**-U**	**-U-M**	**-U**	**-U-M**	**-U**	**-U-M**
**pMET25-Lsm811**	-	+++	+	++	-	++	-	-	-	-
**pGFP-N-Lsm818**	-	+	-	+	-	-	n.t.	n.t.	n.t.	n.t.
**pGFP-C-Lsm818**	-	+++	-	+++	-	+++	-	-	-	-
**pMET25-Lsm818**	-	+++	+	+++	-	+++	-	+	-	-
**pGFP-N-Lsm188**	+	+++	+	+++	+	+++	-	+	-	-
**pGFP-C-Lsm188**	++	+++	++	+++	++	+++	-	+	-	-
**pMET25-Lsm188**	+	+++	+	+++	+	+++	-	+	-	+
**pGFP-N-Lsm18ΔC**	n.t.	-	n.t.	-	n.t.	-	n.t.	-	n.t.	n.t.
**pGFP-C-Lsm18ΔC**	n.t.	-	n.t.	-	n.t.	-	n.t.	-	n.t.	n.t.
**pMET25-Lsm18ΔC**	n.t.	-	n.t.	-	n.t.	-	n.t.	-	n.t.	n.t.
**pGFP-N-Lsm81ΔC**	n.t.	-	n.t.	-	n.t.	-	n.t.	-	n.t.	n.t.
**pGFP-C-Lsm81ΔC**	n.t.	-	n.t.	-	n.t.	-	n.t.	-	n.t.	n.t.
**pMET25-Lsm81ΔC**	-	+	-	+	-	+	n.t.	-	n.t.	n.t.
**pGFP-N-LsmΔN11**	n.t.	-	n.t.	-	n.t.	-	n.t.	-	n.t.	n.t.
**pGFP-C-LsmΔN11**	n.t.	-	n.t.	-	n.t.	-	n.t.	-	n.t.	n.t.
**pMET25-LsmΔN11**	-	++	-	+	-	-	n.t.	-	n.t.	n.t.
**pGFP-N-LsmΔN88**	++	+++	++	+++	+	+++	-	+	n.t.	n.t.
**pGFP-C-ΔN88**	++	+++	++	+++	+	++	-	-	n.t.	n.t.
**pMET25-LsmΔN88**	++	+++	++	+++	++	+++	n.t.	++	n.t.	-
**pGFP-N-LsmΔN1ΔC**	n.t.	-	n.t.	-	n.t.	-	n.t.	-	n.t.	n.t.

Plasmid	Viability in MPS11 (*lsm8Δ*) background									
	18ºC		23ºC		30ºC		36ºC		37ºC	
	-U	-U-M	-U	-U-M	-U	-U-M	-U	-U-M	-U	-U-M
**pGFP-C-LsmΔN1ΔC**	n.t.	-	n.t.	-	n.t.	-	n.t.	-	n.t.	n.t.
**pMET25-LsmΔN1ΔC**	n.t.	-	n.t.	-	n.t.	-	n.t.	-	n.t.	n.t.
**pGFP-N-LsmΔN8ΔC**	n.t.	-	n.t.	-	n.t.	-	n.t.	-	n.t.	n.t.
**pGFP-C-LsmΔN8ΔC**	n.t.	-	n.t.	-	n.t.	-	n.t.	-	n.t.	n.t.
**pMET25-LsmΔN8ΔC**	n.t.	-	n.t.	-	n.t.	-	n.t.	-	n.t.	n.t.
**pGFP-N-LsmΔN18**	n.t.	-	n.t.	-	n.t.	-	n.t.	-	n.t.	n.t.
**pGFP-C-LsmΔN18**	n.t.	-	n.t.	-	n.t.	-	n.t.	-	n.t.	n.t.
**pMET25-LsmΔN18**	n.t.	-	n.t.	-	n.t.	-	n.t.	-	n.t.	n.t.
**pGFP-N-LsmΔN81**	n.t.	-	n.t.	-	n.t.	-	n.t.	-	n.t.	n.t.
**pGFP-C-LsmΔN81**	n.t.	-	n.t.	-	n.t.	-	n.t.	-	n.t.	n.t.
**pMET25-LsmΔN81**	n.t.	-	n.t.	-	n.t.	-	n.t.	-	n.t.	n.t.

Table S3. Viability at non-permissive temperature of *lsm1Δ* expressing mutant Lsm1 proteins

Plasmids were transformed into AEMY25 and tested for growth on SD-Ura and SD-Ura-Met at 36 and 37˚C. Amount and rate of growth were compared to pGFP-N-LSM1 and pGFP-N-FUS and given a score: ++++ for growth like the positive control, – for background growth and +++, ++ or + for anything in between. n.t. = not tested

Viability in AEMY25 (*lsm1Δ*) background				
**Plasmid**	**36ºC**		**37ºC**	
	**-U**	**-U-M**	**-U**	**-U-M**
**pGFP-N-Lsm1**	++++	++++	++++	++++
**pGFP-C-Lsm1**	++	+++	-	++
**pMET25-Lsm1**	++++	++++	+++	++++
**pGFP-N-Lsm881**	-	-	-	-
**pGFP-C-Lsm881**	-	-	-	-
**pMET25-Lsm881**	-	-	-	-
**pGFP-N-Lsm118**	+	+++	-	+
**pGFP-C-Lsm118**	-	+++	-	+
**pMET25-Lsm118**	+	+++	-	++
**pGFP-N-Lsm1ΔC**	-	++	-	+
**pGFP-C-Lsm1ΔC**	-	-	-	-
**pMET25-Lsm1ΔC**	n.t.	n.t.	n.t.	n.t.
**pGFP-N-Lsm181**	n.t.	-	-	-
**pGFP-C-Lsm181**	-	-	-	-
**pMET25-Lsm181**	++	++	-	+
**pGFP-N-Lsm811**	-	-	-	-
**pGFP-C-Lsm811**	++	++	-	+
**pMET25-Lsm811**	-	-	-	-
**pGFP-N-Lsm818**	-	-	-	-
**pGFP-C-Lsm818**	-	-	-	-
**pMET25-Lsm818**	-	-	-	-
**pGFP-N-Lsm188**	-	-	-	-
**pGFP-C-Lsm188**	-	-	-	-
**pMET25-Lsm188**	-	-	-	-
**pGFP-N-Lsm18ΔC**	-	-	-	-
**pGFP-C-Lsm18ΔC**	-	-	-	-
**pMET25-Lsm18ΔC**	-	-	-	-
**pGFP-N-Lsm81ΔC**	-	-	-	-
**pGFP-C-Lsm81ΔC**	-	-	-	-
**pMET25-Lsm81ΔC**	+	+	-	-
**pGFP-N-LsmΔN11**	-	-	-	-

Viability in AEMY25 (*lsm1Δ*) background				
**Plasmid**	**36ºC**		**37ºC**	
	**-U**	**-U-M**	**-U**	**-U-M**
**pGFP-C-LsmΔN11**	-	-	-	-
**pMET25-LsmΔN11**	+	+	-	-
**pGFP-N-LsmΔN88**	-	-	-	-
**pGFP-C-ΔN88**	-	-	-	-
**pMET25-LsmΔN88**	-	-	-	-
**pGFP-N-LsmΔN18**	-	-	-	-
**pGFP-C-LsmΔN18**	-	-	-	-
**pMET25-LsmΔN18**	++	++	-	-
**pGFP-N-LsmΔN81**	-	-	-	-
**pGFP-C-LsmΔN81**	-	-	-	-
**pMET25-LsmΔN81**	-	-	-	-
**pGFP-N-LsmΔN1ΔC**	-	-	-	-
**pGFP-C-LsmΔN1ΔC**	-	-	-	-
**pMET25-LsmΔN1ΔC**	++	++	-	-
**pGFP-N-LsmΔN8ΔC**	-	-	-	-
**pGFP-C-LsmΔN8ΔC**	-	-	-	-
**pMET25-LsmΔN8ΔC**	-	-	-	-

References

[1] Niedenthal RK, Riles L, Johnston M, & Hegemann JH (1996) Green fluorescent protein as a marker for gene expression and subcellular localization in budding yeast. *Yeast*, **12,** 773-786.

[2] Mumberg D, Muller R, & Funk M (1995) Yeast vectors for the controlled expression of heterologous proteins in different genetic backgrounds. *Gene*, **156,** 119-122.

[3] Hu J, Xu Y, Schappert K, Harrington T, Wang A, Braga R, Mogridge J, & Friesen JD (1994) Mutational analysis of the PRP4 protein of Saccharomyces cerevisiae suggests domain structure and snRNP interactions. *Nucleic Acids Res.*, **22,** 1724-1734.

[4] Reijns MAM, Alexander RD, Spiller MP, & Beggs JD (2008) A role for Q/N-rich aggregation-prone regions in P-body localization. *J Cell Sci*, jcs.

[5] Spiller MP, Boon KL, Reijns MAM, & Beggs JD (2007) The Lsm2-8 complex determines nuclear localization of the spliceosomal U6 snRNA. *Nucleic Acids Res.*, **35,** 923-929.

[6] Spiller MP, Reijns MA, & Beggs JD (2007) Requirements for nuclear localization of the Lsm2-8p complex and competition between nuclear and cytoplasmic Lsm complexes. *J Cell Sci*, **120,** 4310-4320.

[7] Teixeira D, Sheth U, Valencia-Sanchez MA, Brengues M, & Parker R (2005) Processing bodies require RNA for assembly and contain nontranslating mRNAs. *RNA*, **11,** 371-382.

[8] Tollervey D & Mattaj IW (1987) Fungal small nuclear ribonucleoproteins share properties with plant and vertebrate U-snRNPs. *EMBO J.*, **6,** 469-476.

[9] Sambrook J & Russell DW (2001) *Molecular cloning*. Cold Spring Harbor Laboratory Press, Cold Spring Harbor, NY.

Fig. S1

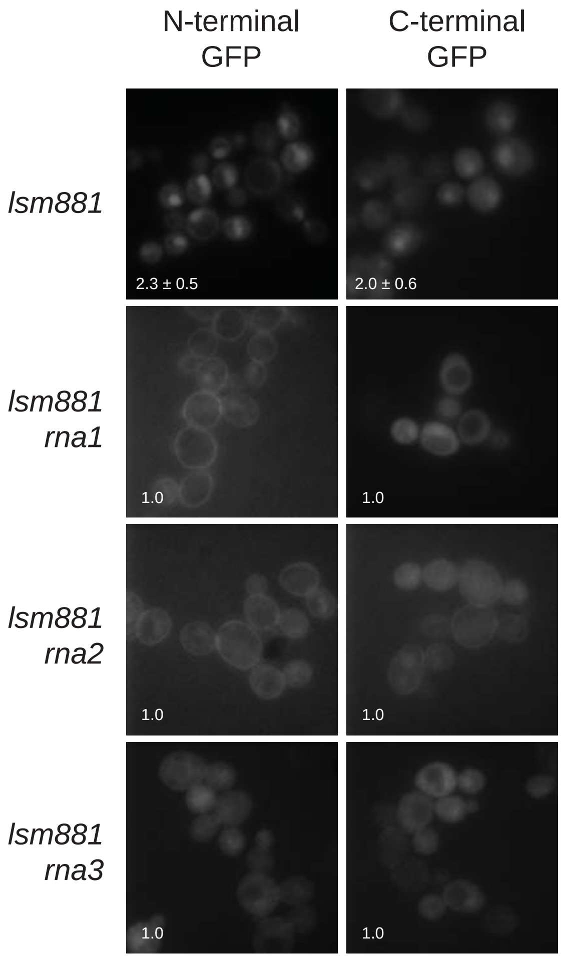
N-terminal GFP
C-terminal GFP
lsm881
lsm881 rna1
lsm881 rna2
lsm881 rna3
2.3 ± 0.5
2.0 ± 0.6
1.0
1.0
1.0
1.0
1.0
1.0

Fig. S2

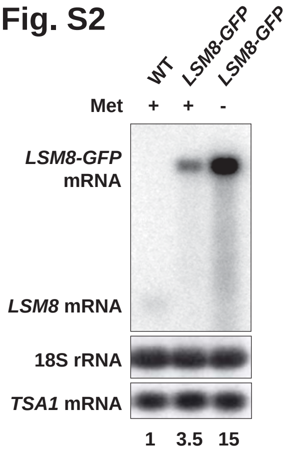

WT
LSM8-GFP
LSM8-GFP
Met
+
+
-
LSM8-GFP mRNA
LSM8 mRNA
18S rRNA
TSA1 mRNA
1
3.5
15

Fig. S3

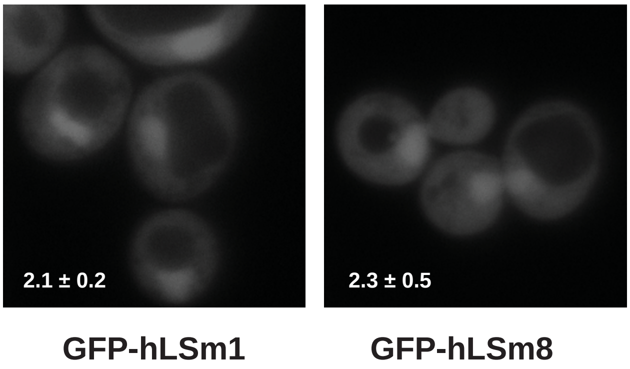

Fig. S1. Effects of mutations in putative RNA binding residues of Lsm8p on nuclear localisation of Lsm881p. MPS26 was transformed with plasmids pMR70, pMR84, pMR73, pMR74, pMR75, pMR95, pMR96 and pMR97 (See Table S1 for plasmid descriptions). Cells were grown in SD-Ura(-Met) and localisation was examined in live cells during log phase growth. Intensities of nuclear and cytoplasmic signals were measured by ImageJ 1.38w and the average ratios of nuclear/cytoplasmic signals are indicated with each image. Where no nuclear accumulation was detected, a ratio of 1.0 is given.

Fig. S2. Comparison of *LSM8-GFP* (expressed from P_{MET25}) and native *LSM8* transcript levels. BMA38a (WT) was transformed with pGFP-C-FUS, MPS11 (*lsm8Δ*) was transformed with pGFP-C-LSM8. Cells were grown in SD-Ura with or without 1 mM methionine. Total RNA was extracted and used for Northern analysis, probing for *LSM8*, and loading controls 18S rRNA and *TSA1* mRNA. Relative levels of *LSM8* mRNA (WT = 1) are indicated. To analyse the level of *LSM8* mRNA, five µg of total RNA, isolated as previously described [8], was glyoxyl denatured and resolved on a standard 1.2% agarose gel [9]. RNA was transferred to Hybond-N+ membrane by passive transfer overnight. This membrane was hybridised with a single-stranded DNA probe covering the entire *LSM8* open reading frame, labelled by 35 PCR cycles in the presence of reverse primer and [α-^{32}P] dCTP. Equal loading was tested for by hybridisation with end-labelled oligonucleotide probes against TCA1 mRNA (GGAGTATTCGGAGTCAGTGGAGGCGAAAAGAACT) or 18S rRNA (CATGGCTTAATCTTTGAGAC). Bands were quantified using a STORM 860 scanner and ImageQuant software (Molecular Dynamics).

Fig. S3. Human LSm1 and LSm8 proteins accumulate in the nuclei of budding yeast cells. BMA38a was transformed with pMR186 or pMR187, grown in SD-Ura-Met and localisation of GFP-hLSm1 or GFP-hLSm8 was examined in live cells during log phase growth. Intensities of nuclear and cytoplasmic signals were measured by ImageJ 1.38w and the average ratios of nuclear/cytoplasmic signals are indicated with each image.
